# Effectiveness of an integrated approach for workplace health promotion on lifestyle of employees: results of a cluster randomized controlled trial

**DOI:** 10.1186/s12889-025-24522-1

**Published:** 2025-10-14

**Authors:** Denise J.M. Smit, Sandra H. van Oostrom, Josephine A. Engels, Allard J. van der Beek, Karin I. Proper

**Affiliations:** 1 National Institute for Public Health and the Environment, Center for Prevention, Lifestyle and Health, Bilthoven, The Netherlands; 2 Department of Public and Occupational Health, Amsterdam UMC, Amsterdam Public Health Research Institute, Amsterdam, The Netherlands; 3 Occupation & Health Research Group, HAN University of Applied Sciences, Nijmegen, the Netherlands

**Keywords:** Integrated approach, Simple lifestyle indicator questionnaire, Worksite health promotion, Cluster-randomized controlled trial, Health behaviors

## Abstract

**Background:**

Evidence for the effectiveness of workplace health promotion programs (WHPPs) is small to moderate. More impact can be expected from an integrated WHPP, including activities at the individual and organizational levels. Since evidence regarding the effectiveness of integrated WHPPs is scarce, the aim of this study was to evaluate the effect of an integrated WHPP on the lifestyle of employees.

**Methods:**

A two-armed cluster randomized controlled trial with measurements at baseline and at six and twelve months of follow-up was conducted. The intervention consisted of health promotion activities aimed at two (out of six) health behaviors, targeting the individual and organizational levels. The main outcome was an overall lifestyle-score measured using twelve items from the Simple Lifestyle Indicator Questionnaire. The secondary outcome measures were six separate health behaviors, i.e. physical activity, nutrition, mental balance, smoking, alcohol consumption, and sleep. Intervention effects at six and twelve months were analyzed by conducting either longitudinal linear or (ordinal) logistic multilevel analyses, or generalized estimating equations.

**Results:**

A total of 173 employees from four Dutch organizations participated. No effect was observed for overall lifestyle. The consumption of sugary drinks was higher over time (OR: 2.4, 95%CI: 1.1–5.4) and after twelve months of follow-up (OR: 2.9, 95%CI: 1.03–8.0) for the intervention condition compared to the control condition.

**Conclusions:**

The integrated WHPP was not effective in improving the lifestyle of employees. The short duration of employees’ exposure to activities, poor implementation (i.e., not meeting the criteria of the integrated WHPP), and the minimal implemented activities may explain the absence of effect.

**Trial registration:**

LTR (onderzoekmetmensen.nl), NL9526. Registered on 3 June 2021.

**Supplementary Information:**

The online version contains supplementary material available at 10.1186/s12889-025-24522-1.

## Background

In the past decades, workplace health promotion programs (WHPPs) have often been deployed to improve the lifestyle and health of employees and their effectiveness has been studied extensively [1–6]. Nevertheless, evidence on the effectiveness of WHPPs is small to moderate and the effects are not always lasting [3, 5, 7]. One of the causes might be that a majority of the WHPPs targets the individual level only [3, 5, 8]. However, when promoting a healthy lifestyle, the focus should not be solely on the individual. Healthy behavior is a combination of both conscious and nonconscious choices [9]. Conscious choices might be affected by providing activities focused on the individual level, e.g., information provision, counseling and workshops. Whereas nonconscious choices can be influenced through the environment by mitigating barriers and increasing opportunities for healthy behavior [9]. For example, activities at the organizational level, such as reducing the availability of soft drinks or providing equipment to stimulate active meetings, could be implemented. Hence, in addition to activities targeting the individual, a work environment that supports healthy choices contributes to the success of the adoption of healthy lifestyle behaviors [6, 9, 10]. The importance of a supportive work environment has been confirmed in a recent study, which showed that colleague encouragement and colleague behavior play a role in the participation rate of WHPPs, i.e. the use of healthy menus and sport facilities [11]. Additionally, support from supervisors has been found to be important in improving the uptake of WHPPs [12–14]. WHPPs often focus on one health behavior, e.g., through a physical exercise or mindfulness intervention [3, 5]. However, health behaviors are often intertwined. Hence, targeting multiple health behaviors within one WHPP might yield greater effects [2]. For instance, to reduce body weight, a combination of healthy diet and sufficient physical activity might be most effective, and poor nutrition might affect sleep quality [2, 4, 15].

A WHPP that targets both the individual and organizational levels and multiple health behaviors simultaneously is considered an integrated WHPP [16]. The Lombardy Workplace Health Promotion Network (LWHPN) is an example of an integrated WHPP, which is recognized as a good practice in the occupational setting in the European Joint Action CHRODIS [16, 17]. In the LWHPN, participating organizations received a catalogue with health promoting activities for multiple health behaviors at both the organizational and individual levels. Organizations then chose which activities they intended to implement in their organization and thus composed their own tailored integrated WHPP. The results from a one-year pilot study were promising and implementation of the program in practice was successful [16, 18, 19]. Significant positive effects for smoking cessation and fruit and vegetable intake were observed [19]. In Andalusia, Spain, a similar program based on the LWHPN has also been successfully implemented [20]. Although no significant changes in sweet consumption or PA were found after nine months in this Spanish non-controlled study, positive tendencies were observed in organization-segmented before-after analyses [21]. The researchers did not provide an explanation for the difference in effect between the approach in Andalusia and Lombardy.

Currently, scientific evidence, using robust evaluation methods, concerning the effectiveness of such integrated WHPPs is scarce. To illustrate, both the LWHPN and the Andalusian integrated WHPP were evaluated by conducting non-randomized and or non-controlled studies. Therefore, we developed an integrated WHPP based on the LWHPN, tailored to the needs of employees and employers in the Netherlands and planned to evaluate its effectiveness [22]. Since cultural differences can influence the content of the program—which is tailored to the specific needs of employees within the organization—as well as its implementation and effectiveness, it is crucial to study such integrated WHPPs in diverse cultural contexts. Hence, the aim of this study was to evaluate the effect of the developed integrated WHPP on the lifestyle of Dutch employees, by conducting a cluster randomized controlled trial, the golden standard to assess the effectiveness of an intervention. Therefore, our study contributes to the body of evidence regarding the effectiveness of integrated WHPPs in various occupational and cultural contexts.

## Methods

### Study design and recruitment

#### Recruitment of organizations

The effectiveness of the integrated WHPP was evaluated in a two-armed cluster randomized controlled trial (C-RCT), with follow-up measurements at six and twelve months, which was conducted between January 2022 and March 2024. Four organizations in different occupational sectors (i.e. two educational organizations, an assurance, tax and consulting organization and a retail organization) participated in the C-RCT (Additional file 1). Within one of the educational organizations, only the ICT and facility departments participated. The participating organizations were recruited through the networks of the research team, coworkers and branch specific networks. 62 organizations showed interest in participating in the study, and four of these eventually did participate in the study. Organizations were eligible for participation if they had at least 200 employees and had not yet implemented a WHPP similar to the WHPP under study, i.e., WHPPs with a focus on both the organizational and individual levels and/or activities within multiple health behaviors. Of the 58 organizations that did not participate in the study, five already implemented a program similar to the integrated WHPP. Eight others were to small (i.e., less than 200 employees). The other 45 organizations had various reasons not to participate, such as staff turnover, impact of COVID-19 (specifically in healthcare organizations), or other priorities. The participating organizations implemented the integrated WHPP voluntarily, driven by their own interest in promoting healthy lifestyles at work, rather than out of a legal obligation within their occupational health and safety management.

The Medical Ethical Committee of VU University Medical Center (VUmc, Amsterdam, the Netherlands) approved the study protocol (2021.0402). The trial is registered in the Dutch Trial Register (LTR) under the number NL9526. Details on the development, sample size calculation, methods and outcome measures have been described in a protocol paper [22]. We adhered to the CONSORT checklist for reporting our data analysis procedures (Additional file 2) [23].

#### Recruitment of participants

Employees within the participating organizations were informed about the study through different communication channels (e.g., e-mails, flyers, newsletters, and/or online communication apps, such as Microsoft Teams, and/or internal websites). Subsequently, employees were invited for an information session at the workplace or online to provide more detailed information about the study. Additionally, the employer emphasized the importance of this study and encouraged employees to participate. A QR code was placed on all recruitment materials and distributed during the online information sessions. Those interested in participating in this study received an information letter, eligibility checklist and informed consent at home by post.

The inclusion criteria for participants were: working for the participating organization for 12 or more hours per week, a good understanding of the Dutch language and an employment contract that either lasted until the final measurement or was to be extended to it. The exclusion criteria were: being on sick leave for more than four weeks or being pregnant.

**Fig. 1:**
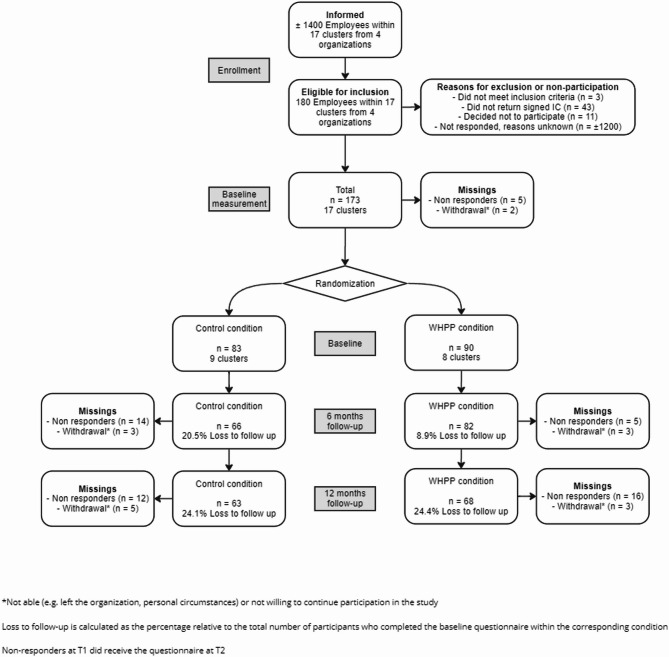
Flowchart inclusion and (non-) response for the different measurements. Not able (e.g. left the organization, personal circumstances) or not willing to continue participation in the study. Loss to follow-up for each condition was calculated as the percentage relative to the total number of participants who completed the baseline questionnaire within the corresponding condition. Non responders at six months of follow-up did receive the questionnaire after twelve months of follow-up

Figure 1 shows the flow of participants throughout the trial. 

At the time of recruitment approximately 1,400 workers were employed within 17 clusters of the four participating organizations. Among these employees, information about the study was disseminated by the employer. In total, 180 of the employees who responded to the call and were willing to participate, were eligible for inclusion in the current study. Others were either excluded or did not return a signed informed consent. Approximately 1,200 employees did not respond to the call for participation. This resulted in a response rate of 12.8%.

The baseline questionnaire was completed by 173 participants. The six-month follow-up questionnaire was sent to 171 participants, as two participants dropped out prior to the second measurement. The questionnaire was completed by 148 participants (86% of the baseline participants). A total of 163 participants received the twelve-month follow-up questionnaire, due to eight dropouts between the six- and twelve-month follow-up measurements. The questionnaire was completed by 131 participants (76% of the baseline participants). All participants who completed at least one follow-up questionnaire were included in the longitudinal analysis (*n* = 153).

### Randomization, blinding, and sample size calculation

Block randomization was carried out by two independent researchers at the cluster level after the baseline measurement using a computer program, in which one of the independent researchers applied varying block sizes [24]. Clusters were composed based on working locations, as an attempt to reduce contamination between the control and intervention conditions. Clusters in the intervention condition were instructed to implement the integrated WHPP within six months after randomization. Clusters in the control condition were placed on a waiting list and could implement the intervention after twelve months of follow-up. The researcher involved in data processing and analyses was blinded to group allocation. In the proposed study, 264 employees were necessary at twelve months of follow-up to obtain a power of 80% to statistically demonstrate a 10% lifestyle improvement, as measured by the Simple Lifestyle Indicator Questionnaire.

### The intervention

The integrated WHPP consisted of a catalogue of health promoting activities and an implementation plan that assisted in successfully implementing the health promoting activities according to the criteria of the integrated WHPP. The integrated WHPP was developed with input from the target group (Dutch employees and employers) following the Map of Adaptation Process (MAP) [22, 25]. The MAP consists of five steps: (1) assessment of relevant health behaviors, potential barriers and facilitators for implementation, potential activities to be included in the catalogue and the formulation of the criteria of the integrated WHPP, (2) selection of the final content of the catalogue, (3) preparation of the catalogue of implementation, (4) pilot test of the feasibility and comprehensiveness of the implementation plan and (5) implementation of the program. Further details are described in the protocol paper [22].

#### Catalogue

The catalogue included evidence- and/or practice-based health promoting activities for six health behaviors at both the individual and organizational levels, including activities for employees working from home. The catalogue was based on the CHRODIS + toolkit [26]. The health behaviors included were: physical activity, nutrition, mental balance (stress, work-home balance, relaxation), smoking, alcohol consumption, and sleep. Examples of activities include exercise activities, healthy nutrition in the company restaurant, active work stations or stress management courses (Additional file 3). The individual level comprises two domains: (1) screening and support, which focuses on the identification of lifestyle-related issues and support in addressing these issues, and (2) information and education, in which stimulating awareness about the importance of a healthy lifestyle was key. The organizational level was also subdivided into two domains: (3) adjustments in the social, digital or physical work environment to support a healthy lifestyle and (4) policy adjustments to facilitate and encourage a healthy lifestyle. To meet the criteria of the integrated WHPP, activities had to be implemented on at least two different health behaviors and on both the individual and organizational levels for each health behavior. These criteria were established by the researchers, and are based on the definition for an integrated approach of the Lombardy WHP Network and the definition of other Dutch integrated health promotion programs developed by the National Institute of Public Health and the Environment, Center of Healthy Living [22].

#### Implementation plan

The implementation plan provided a step-by-step plan, which was developed based on barriers to and facilitators of the participation in and implementation of WHPPs according to employees and employers, respectively [14, 27]. A working group consisting of HR professionals, employees and supervisors was composed within each organization. This working group was responsible for a needs assessment and the selection and implementation of activities. The contact person within each organization (often an HR professional or someone involved in WHP) composed the working group by either an open call for employees and supervisors to apply or by personally approaching colleagues.

### Outcome measures

The participants received an online questionnaire at baseline and at six and twelve months of follow-up. All outcome measures pertain to the individual participant.

#### Primary outcome

The primary outcome was the overall lifestyle of employees, which was measured via the validated Simple Lifestyle Indicator Questionnaire (SLIQ) [22, 28, 29]. The SLIQ provides a global lifestyle score and consists of five components: nutrition (3 questions), physical activity (3 questions), alcohol consumption (3 questions), smoking status (2 questions), and stress (1 question). For each lifestyle component in the SLIQ, a score of 0–2 was assigned yielding a total score of 0–10 for the overall lifestyle score, where 0 represents the most unhealthy lifestyle and 10 represents the healthiest lifestyle possible. The SLIQ is also proven to be sensitive to change. A SLIQ score that increases over time correlates with a healthier lifestyle, as measured by a combination of a decrease in weight and self-reported improved lifestyle. Additionally, a SLIQ score which decreases correlates with a less healthy lifestyle [30].

#### Secondary outcomes

The secondary outcome measures involved the separate health behaviors included in the catalogue of the integrated approach, i.e. physical activity, nutrition (sugary drinks and snacks), mental balance (stress, work-home balance and need for recovery), sleep (quality and quantity), smoking and alcohol consumption. All health behaviors were considered as it was not known beforehand which health behaviors would be targeted by the organizations.

*Physical activity* was measured using the validated Short QUestionnaire to ASsess Health-enhancing physical activity (SQUASH) [31]. The SQUASH questionnaire measures the amount of time spent, during a regular week in the past month, in four different physical activity domains: commuting, occupational, household, and leisure time. The outcome measures included in the current study were: minutes of light physical activity (LPA), moderate physical activity (MPA) and vigorous physical activity (VPA) per week; these measures encompass time spent in all four physical activity domains.

For *nutrition*, questions referred to the consumption of sugary drinks and small and large snacks, both sweet, savory and deep-fried per week, during a regular month [32]. With respect to the consumption of sugary drinks, questions were answered on a five-point scale (i.e., < 1, 1–6, 7–13, 14–20 and ≥ 21 sugary drinks per week). This variable was dichotomized (i.e., < 1 vs. ≥ sugary drinks per week) by merging the four highest categories, as the number of participants in these categories was low. Questions about five types of snacks, i.e., large and small sweet snacks, large and small savory snacks and fried snacks, were answered on a six-point scale (i.e. <1, 1, 2–3, 4–6 snacks per week, 1 per day and ≥ 2 per day). For the analysis, the five variables for snacks were combined into two variables, i.e., large snacks and small snacks, each with three categories. This process was performed in three steps. First, the categories were converted to the same unit, namely, the number of snacks per week (i.e., 0, 1, 3, 5, 7 or 14 snacks per week). Second, the values for large savory, large sweet and fried snacks were summed, as were the values for small savory and small sweet snacks. Resulting in two continuous snack variables, i.e., large snacks and small snacks. Third, tertiles were generated based on baseline values of the large (0–2, 2–4 and 4–13) and small (0–4, 4–6 and6-28) snacks, which were then used to establish three categories for each variable for the statistical analysis.

With regard to *mental balance*, data about stress, work-life balance and need for recovery were collected. A subscale of the short version of the Depression Anxiety and Stress Scale (DASS-21) was used [33]. This included seven statements to assess overall stress during the past week. The answers were summed to a total score ranging from 0 to 21 and then converted into five categories: normal, mild, moderate, severe and extremely severe stress. Due to low number of participants in the latter three categories, they were merged into one category, i.e., moderate to severe stress. The short version of the negative work-home interference scale of the Survey Work-home Interference Nijmegen (SWING) was used to measure work-life balance [34, 35]. The extent to which work-life negatively interferes with home-life was assessed based on four items with a four-point scale. The scores of the four items were summed and averaged, leading to an overall score ranging from 0 to 3, in which 3 is the most negative work-home interference possible. A subscale of the Questionnaire on the Experience and Evaluation of Work was used to measure the need for recovery [36, 37]. This scale consists of eleven statements that had to be answered as either yes or no. A score of 0 was assigned to the positive answer and 1 to the negative answer. The sum of the items was then standardized to a score ranging between 0 and 100, in which a score of 100 was the highest need for recovery. All the questionnaires were found to be valid and reliable [33, 35, 36, 38, 39].

*Sleep* quality and quantity in the past four weeks as perceived by the participants were assessed using the validated Medical Outcomes Study Sleep scale (MOS-SS) [40]. Sleep quality comprised sleep disturbance and somnolence and were measured with four and three items, respectively. These items were scored on a six-point scale and converted to a score between 0 and 100. A higher score indicated greater perceived sleep disturbance or greater somnolence. Sleep quantity was assessed by the average hours of sleep per night in the past four weeks.

*Smoking* status, yes or no, was assessed by the first question regarding smoking included in the SLIQ, i.e., are you a smoker? Similarly, questions regarding *alcohol consumption* included in the SLIQ were used to calculate the total consumption of alcoholic beverages during an average week. To do so, the average number of beers, wine and spirits consumed in one week were summed.

#### Covariates

Data about sex, age, educational level, self-reported chronic diseases, self-perceived health, measured using the RAND-36, physical job intensity and working situation i.e., working from home or at the workplace fulltime or parttime, were collected at baseline [41].

#### Process evaluation measures

A mixed-methods process evaluation was conducted alongside the trial to gain insight into the implementation of the integrated WHPP. Details on the methodology and full findings of the process evaluation are reported elsewhere [42]. A brief summary of the main findings is provided in additional file 4.

### Statistical analysis

Descriptive analyses were conducted for both conditions separately and for the total study population. All analyses were performed according to the intention-to-treat principle; thus, effect outcomes were evaluated regardless of whether organizations met the criteria of the integrated WHPP and regardless of the participation levels of employees. This approach aligns with our aim to evaluate the impact of implementing an integrated WHPP as a whole, independent of the specific behavioral themes prioritized by organizations. A detailed overview of the themes addressed and behavior-specific outcomes is reported in a separate publication [43].

For the analysis of the primary outcome measure over time and at six and twelve months of follow-up, a linear multilevel analysis with three identified levels (working locations, i.e. the clusters, participants and time) was performed. To assess the effect of the integrated WHPP at six and twelve months, an interaction term for time and condition was added. First, an analysis adjusted for the baseline value of the outcome measure (model 1) was performed to evaluate the differences between the control and intervention conditions over time and at six and twelve months of follow-up. Second, the abovementioned analyses were performed and additionally adjusted for demographic factors (age, sex and educational level) and self-perceived health measured at baseline (model 2). Effect modification was considered for working situation (working from home or at the workplace, fulltime or parttime) measured at baseline, and a *p*-value < 0.1 of the interaction term was used to indicate effect modification.

Analyses of secondary continuous outcomes, i.e. physical activity, need for recovery, work-home balance, alcohol consumption and sleep, were identical. The categorical secondary outcome measures, i.e. snacks (large and small) and stress were analyzed using an ordinal logistic multilevel analysis. Dichotomous outcomes, namely, the consumption of sugary drinks and smoking, were analyzed by conducting a generalized estimating equation (GEE) which was adjusted for the clustering of repeated measures. An additional adjustment for organization was included. The analyses for secondary outcome measures followed the same procedure as mentioned above. To assess differences between drop-outs and participants, t-tests and Fisher tests were conducted; additionally, a Bonferroni correction was performed to account for multiple comparisons. All statistical analyses were performed using Rstudio version 2023.03.1 (lme4, geepack and ordinal packages) [44–47].

## Results

### Demographics

Slightly more than half of the participants (52.6%) were female (Table 1). The mean age of the participants was 43.3 years (SD = 11.9), and 66.7% had a high educational level. More than one third (38.7%) of the participants had one or more self-reported physical or mental health problem(s). A low level of physical load at work was reported by 79.8% of the participants. The average number of working hours per week was 35.7 h (SD = 7.6), and 39.9% worked only at the workplace and not from home.

**Table 1:** Baseline characteristics of participants in the control condition, intervention condition and total study population

Characteristics	Control condition (*n* = 83)	Intervention condition (*n* = 90)	Total (*n* = 173)
Sex, female, n(%)	39 (47.0)	52 (57.8)	91 (52.6)
Age, mean (SD), years	44.7 (11.2)	42.5 (11.6)	43.6 (11.5)
Educational level, n(%)			
* Lower education*	3 (3.6)	6 (6.7)	9 (5.2)
* Moderate education*	24 (28.9)	24 (26.7)	48 (27.7)
* Higher education*	56 (67.5)	60 (66.7)	116 (67.1)
One or more chronic diseases^a^, n(%)	36 (43.4)	31 (34.4)	67 (38.7)
Work			
Working hours per week, mean (SD)	35.6 (7.1)	35.9 (8.0)	35.7 (7.6)
Job intensity, n(%)^bc^			
* Low physical load*	60 (72.3)	78 (86.7)	138 (79.8)
* Light physical load*	22 (26.5)	7 (7.8)	29 (16.7)
* Moderate physical load*	1 (1.2)	5 (5.5)	6 (3.5)
Working from home, n(%)			
* Never*	38 (45.8)	31 (34.4)	69 (39.9)
* Parttime*	43 (51.8)	56 (62.2)	99 (57.2)
* Fulltime*	2 (2.4)	3 (3.3)	5 (2.9)

^a^Self-reported physical or mental health problems

^b^Low physical load: A sedentary occupation. Light physical load: A standing occupation, including walking but not high intensity physical activity. Moderate physical load: An occupation that included occasional heavy lifting

^c^Significant difference (*p*=0.002) between intervention and control condition based on a chi-square test. A significance level of *p *< 0.007 with Bonferroni correction was applied to account for multiple comparisons

Table 2 reports the baseline values of the outcome measures. The mean lifestyle scores at baseline were 7.0 (SD = 1.5) and 7.2 (SD = 1.5), on a scale from 0 to 10 for the control and intervention conditions, respectively. Drop-out analyses did not reveal differences between the drop-outs and participants (Additional files 5 and 6).

**Table 2:** Means and frequencies for the primary and secondary outcome variables at baseline and at six and twelve months of follow-up

	Baseline		Six months follow-up		Twelve months follow-up	
	C (*n* = 83)	I (*n* = 90)	C (*n* = 66)	I (*n* = 81)	C (*n* = 63)^a^	I (*n* = 68)^b^
Overall lifestyle, mean (SD)	7.0 (1.5)	7.2 (1.5)	7.2 (1.5)	7.1 (1.5)	7.1 (1.5)	7.2 (1.4)
Physical activity						
LPA, minutes per week, mean (SD)	2225.1 (1025.8)	2502.4 (834.0)	2176.0 (947.9)	2378.8 (1089.6)	2243.6 (895.1)	2244.4 (1034.6)
MPA, minutes per week, mean (SD)	589.2 (538.5)	445.8 (549.7)	602.1 (477.7)	422.1 (374.2)	663.0 (557.0)	510.0 (479.9)
VPA, minutes per week, mean (SD)	123.4 (158.2)	86.9 (127.9)	121.2 (138.5)	92.0 (131.0)	124.5 (171.2)	76.9 (114.7)
Nutrition						
Sugary drinks per week, *n*(%)						
* < 1 per week*	39 (47.0)	47 (52.2)	33 (50.0)	39 (48.1)	32 (52.5)	32 (47.1)
* ≥ 1 per week*	44 (53.0)	43 (47.8)	33 (50.0)	42 (51.9)	29 (47.5)	36 (52.9)
Large snacks^c^ per week, *n*(%)						
* 0–2 per week*	32 (38.6)	44 (48.9)	25 (37.9)	33 (40.7)	25 (41.0)	27 (40.7)
* 2–4 per week*	29 (34.9)	21 (23.3)	23 (34.8)	31 (38.3)	21 (34.4)	23 (33.8)
* 4–13 per week*	22 (26.5)	25 (27.8)	18 (27.3)	17 (21.0)	15 (24.6)	18 (26.5)
Small snacks^d^ per week, *n*(%)						
0–4 *per week*	35 (42.2)	34 (37.8)	28 (42.4)	36 (44.4)	27 (44.3)	30 (44.1)
4–6 *per week*	19 (22.9)	28 (31.1)	21 (31.8)	20 (24.7)	14 (23.0)	17 (25.0)
6–28 *per week*	29 (34.9)	28 (31.1)	17 (25.8)	25 (30.9)	20 (32.8)	21 (30.9)
Mental balance						
Perceived stress, *n*(%)						
* Normal*	62 (74.7)	65 (72.2)	56 (84.8)	59 (72.8)	51 (83.6)	48 (71.6)
* Mild*	13 (15.7)	12 (13.3)	6 (9.1)	12 (14.8)	6 (9.8)	11 (16.4)
* Moderate to severe*	8 (9.6)	13 (14.5)	4 (6.1)	10 (12.4)	4 (6.6	8 (12.0)
NFR, mean (SD)	30.4 (30.4)	38.2 (31.8)	28.5 (29.6)	33.6 (30.4)	27.6 (28.3)	37.4 (31.4)
Work-life balance, mean (SD)	0.8 (0.6)	0.8 (0.6)	0.8 (0.6)	0.8 (0.5)	0.8 (0.5)	0.7 (0.6)
Sleep						
Sleep disturbance, mean (SD)	25.0 (14.3)	30.7 (19.9)	26.9 (15.4)	30.5 (18.1)	25.8 (14.1)	31.8 (19.9)
Somnolence, mean (SD)	21.8 (15.6)	22.0 (14.4)	23.1 (16.2)	25.1 (15.0)	23.3 (16.3)	22.6 (14.4)
Hours of sleep per night, mean (SD)	7.3 (0.8)	6.9 (1.0)	7.0 (0.9)	6.8 (1.2)	7.1 (0.8)	6.8 (1.2)
Smoking						
Smoking status, yes, n(%)	12 (14.5)	1 (1.1)	9 (13.6)	3 (3.7)	7 (11.1)	1 (1.5)
Alcohol						
Alcohol consumption per week, mean (SD)	4.3 (4.7)	4.1 (4.9)	5.4 (5.9)	5.5 (6.2)	3.6 (4.3)	3.8 (4.5)

*C* Control condition, *I* Intervention condition, *LPA* Light Physical Activity, *MPA* Moderate Physical Activity, *VPA* Vigorous Physical Activity, *NFR* Need for recovery

^a^*N* = 2 participants did not fully complete the questionnaire

^b^*N* = 1 participant did not fully complete the questionnaire

^c^Large snacks: sweet, savory and fried

^d^Small snacks: Sweet and savory

### Effect of the intervention

Table 3 shows the estimated effect sizes of the intervention on the primary and secondary outcome measures over time and at six and twelve months of follow-up. There was no effect modification of the working situation.

**Table 3:** Effect estimates based on model 2 for the primary and secondary outcomes of the WHPP intervention over time and after six and twelve months of follow-up

	6 months	12 months	6–12 months
	β (CI)	β (CI)	β (CI)
Overall lifestyle	−0.2 (−0.5–0.2)	−0.1 (−0.5–0.3)	−0.1 (−0.5–0.2)
Physical activity	**β*(CI)***	**β*(CI)***	**β*(CI)***
LPA minutes per week	147.8 (−156.3–453.6)	−62.5 (−381.6–259.8)	53.3 (−195.1–303.6)
MPA minutes per week	−140.8 (−310.2–29.0)	−107.8 (−282.7–67.6)	−129.5 (−284.9–26.6)
VPA minutes per week	−0.1 (−36.6–35.7)	−22.2 (−59.9–14.9)	−9.9 (−43.6–22.6)
Nutrition	**OR*(CI)***	**OR*(CI)***	**OR*(CI)***
Sugary drinks	2.0 (0.6–6.4)	2.9 (1.03–8.0)^c^	2.4 (1.1–5.4)^c^
Large snacks^a^	0.7 (0.3–1.8)	1.0 (0.4–2.9)	1.0 (0.5–2.3)
Small snacks^b^	0.9 (0.3–3.0)	0.8 (0.2–2.6)	1.0 (0.4–2.7)
Mental balance	**OR/β*(CI)***	**OR/β*(CI)***	**OR/β*(CI)***
Stress (OR)	3.3 (0.9–12.2)	2.8 (0.7–10.8)	3.0 (1.0–9.5)
Need for recovery (β)	1.2 (−6.8–9.3)	3.9 (−4.4–12.3)	2.3 (−5.2–9.9)
Work-life balance (β)	−0.1 (−0.3–0.1)	−0.1 (−0.2–0.1)	−0.1 (−0.2–0.1)
Sleep	**β*(CI)***	**β*(CI)***	**β*(CI)***
Sleep disturbance	0.3 (−3.4–4.0)	1.8 (−2.1–5.8)	1.0 (−2.3–4.2)
Sleep somnolence	3.0 (−0.3–6.2)	−1.3 (−4.8–2.3)	1.1 (−1.8–4.0)
Sleep quantity	−0.2 (−0.6–0.2)	−0.3 (−0.7–0.1)	−0.3 (−0.7–0.1)
Alcohol consumption	**β*(CI)***	**β*(CI)***	**β*(CI)***
Consumptions per week	0.1 (−1–1.1)	0.3 (−0.8–1.4)	0.2 (−0.7–1.1)

Effect estimates based on model 2, adjusted for baseline values of the outcome measure, age, sex, education and perceived health at baseline, were reported

*β * Beta (regression coefficient), *OR* Odds ratio, *CI* 95% Confidence Interval, *LPA* Light Physical Activity, *MPA* Moderate Physical Activity, *VPA* Vigorous Physical Activity, *NFR* Need for recovery

^a^Large snacks: sweet, savory and fried.

^b^Small snacks: sweet and savory. Smoking status was not included in the analysis, as none of the models converged

^c^Statistically significant difference

No statistically significant differences were found in the overall lifestyle of employees between the intervention and control conditions over time or at six months and twelve months of follow-up. With the exception of the consumption of sugary drinks, there were also no intervention effects on any of the secondary outcomes. It appeared that over time and at twelve months of follow-up participants in the intervention condition had 2.4 (95%CI: 1.1–5.4) and 2.9 (95%CI: 1.03–8.0) higher odds of consuming ≥1 sugary drink per week, respectively, compared to participants in the control condition.

## Discussion

### Main findings

The current study revealed no effects of the integrated WHPP on the overall lifestyle score. With respect to secondary outcomes, a greater consumption of sugary drinks in the intervention condition over time and at twelve months of follow-up was observed, compared to the control condition.

### The impact of COVID-19

An important limitation of the current study is that the a priori calculated sample size was not met. The recruitment and start of the intervention study took place during the COVID-19 pandemic, which hindered the recruitment of organizations and participants. Moreover, many organizations could not participate due to a lack of time, other priorities or unfavorable timing. As a result, only 173 employees participated in this study at baseline, instead of the calculated 264 employees. While the small sample size reduced statistical power, the observed small effect size suggests that the intervention had limited impact. This likely reflects implementation issues rather than merely a lack of power. A larger sample might have yielded significance but would not have addressed the underlying implementation challenges. Future research should focus on improving implementation to better assess the intervention’s true effectiveness. At the time of the trial, more employees were working from home than before the COVID-19 pandemic, which might have impacted the recruitment and participation of employees. However, as we conducted a C-RCT this impact would be similar in both the intervention and control condition. We therefore expect that this did not affect the outcome. We also included activities that were suitable for hybrid workers in the catalogue. None of the organizations implemented activities specifically for hybrid workers. As most employees worked at least parttime at the physical working location, we do not expect this to impact the results. Future studies can provide insight into how to sufficiently reach and include hybrid workers for (studies towards) WHPPs.

### Implementation of the integrated WHPP

Two key elements of the integrated WHPP were: 1) the implementation of activities at both the individual and organizational levels for multiple lifestyle themes and 2) the selection and implementation of activities that fit the organization and the needs of employees by a working group, consisting of HR, supervisors and employees [42]. However, based on the results of the process evaluation, it appeared that in particular the implementation of the first key element did not succeed as intended in practice. This was mainly due to the absence of organizational policies regarding vitality and a lack of time reported by the members of the working group [42]. All the organizations implemented activities targeting at least two different health behaviors, but the activities were not on both the individual and organizational level, indicating that the criteria of the integrated WHPP were not met [42]. Consequently, we cannot assess the ‘true’ effect of the integrated WHPP as intended; rather, we examined the effect of the'integrated WHPP as implemented by the organizations'. The fact that the integrated WHPP was not implemented as intended might partially explain the absence of an effect on the primary outcome, i.e., overall lifestyle. As it was unclear beforehand which health behaviors from the catalogue the organizations would target, secondary outcome measures were included for each of the health behaviors in the catalogue. Eventually, two organizations mainly targeted physical activity, one organization nutrition and one organization mental balance. Therefore, an effect for the non-targeted health behaviors was not expected. Although not all organizations targeted for instance, physical activity, all organizations were included in the analysis of that health behavior. This might mitigate the potential effect, therefore, we further investigated and reported the specific effects on the targeted health behaviors in a separate paper [43].

### Characteristics of implemented activities

Three factors related to the implemented activities are potentially attributed to the absence of effects in our study. First, the implementation of simple and minimal activities or adjustments, such as nudges to take the stairs or information messages at an internal website. Second, the low frequency and irregular occurrence of activities, for instance exercise workshops or a one-time tasting event. And third, the short exposure of employees to the activities, as most activities were implemented shortly before the six-month follow-up measurement.

The choice for minimal and easy to implement activities was also observed in a study of a comparable WHPP, in which project leaders selected evidence-based activities from a list based on a needs assessment among employees [48]. Most of the implemented activities in the study of Wierenga et al. (2014) did not require active participation of employees, but included, for instance, free fruit at the workplace or posters. Activities implemented by organizations in the LWHPN required both active (i.e., physical activity events, social events) and passive participation (i.e., healthy offer in the company restaurant, posters about nutrition, smoking and alcohol consumption policies). However, some of these passive activities are more extensive than posters, such as the healthy offer at the company restaurants and policies regarding smoking or alcohol consumption, which might increase effectiveness compared to small passive activities [19]. In the study of Wierenga et al., the researchers stated that low costs and low implementation effort were prioritized over effectiveness when selecting suitable activities.

These minimal activities are positive initial steps, which might create awareness rather than actual behavior change, as behavior change requires a more intensive approach [5, 48, 49]. To promote healthy habits, repetition of the desired behavior in a stable context is an important component [49, 50]. For instance, Kaushal and Rhodes (2015) indicated that to adopt new, healthy physical exercise habits, approximately six weeks of regular exercise work outs were required [51]. The importance of activities with high frequency and regularity has also been highlighted in other studies [5, 12]. Wierenga et al. (2013) reported that activities that occurred once per week were four times more effective in improving nutrition and physical activity health behaviors than activities with a lower weekly frequency [5]. Nöhammer et al. (2010) reported that the regularity of activities could enhance effectiveness [12].

In our study, the short exposure of employees to the activities is a result of the extensive time required for establishing the working group, consequently causing a delay in the implementation of activities. If the amount of time between activities is too long, it might affect the enthusiasm to participate [48]. Thus, the delay in implementation might have affected participation in activities, which in turn can negatively impact effectiveness [52, 53]. To increase effectiveness, extensive activities taking place regularly without delays between separate activities are required. As costs and implementation efforts were important considerations for organizations when selecting activities, it is essential to find a middle ground between activities that are both effective, low-cost and low-effort [48]. Organizations need to have a clear understanding of which activities meet these requirements. The responsibility for implementing more extensive activities is thus shared. While organizations should not only focus on simple activities, they also require additional support to make the right choice. For instance, information about the activities regarding effectiveness, costs and implementation efforts should be explicitly outlined in the catalogue in future studies.

### Comparison with similar interventions

As to the secondary outcomes, our findings are in contrast with results of a previous non-randomized controlled before-after evaluation of the effects of the Lombardy Workplace Health Promotion Network (LWHPN), where the intake of fruit and vegetables and smoking cessation increased significantly after twelve months [19]. Based on a quasi-experimental controlled trial, days of fruit consumption also increased in a study conducted by Wierenga et al. (2014), but no effect was observed for pieces of fruit consumed and physical activity outcome measures [48]. In line with our study, both in the LWHPN study and in the study of Wierenga et al. organizations composed a working group including employees that was instructed to implement activities from a list or catalogue. A difference between our study and the one by Wierenga et al., was that a project leader was appointed in the study of Wierenga et al., who could allocate 16 hours per week to the implementation of activities [48]. Additionally, an external advisor was available, to support implementation. However, an even more active role of this advisor might be required, in assisting organizations to select activities that are both easy to implement and effective. Organizations in the LWHPN received support from the Lombardy Region to which they had to report about the implementation process and planning. This might positively attribute to successful implementation and consequently, the effectiveness of the LWHPN, as a concrete implementation plan has to be developed each year [18]. In our study, organizations received minimal implementation support as the researchers occupied an observational role to assess the ability of organizations to implement the integrated WHPP independently. These differences in implementation and support might attribute to the differences in observed effectiveness between the LWHNP and the integrated WHPP under study. In line with Wierenga et al., implementing the intervention on their own, without support from the researchers, appeared to be challenging for the organizations. Hence, in addition to guidance regarding the selection of activities, more extensive implementation support might be required in the future. Moreover, to enhance feasibility of implementation for organizations, it could be considered to spread out implementation by focusing on different health behaviors each year while maintaining existing activities. This is also consistent with the LWHPN, where organizations can receive a vignette upon implementing new activities that target different health behaviors annually while retaining existing activities [18].

### Other limitations

With regard to the consumption of sugary drinks, surprisingly, participants in the intervention condition were more than two times more likely to consume more than one sugary drink per week over time, compared to the control condition. At each measurement moment, the percentage of employees in both conditions who consumed more than one sugary drink was approximately 50%. Indicating that still a large percentage consumed less than one sugary drink per week. Moreover, the four categories with the highest number of sugary drinks had to be merged because of the low number of participants in these categories. This implies that, overall, the consumption of sugary drinks among the participants was low. Thus leaving little room for improvement due to a ceiling effect. The consumption of sugary drinks was targeted by one organization, which replaced sodas with healthy syrups. It is plausible that employees also considered these healthy syrups to be sugary drinks and thus reported them as such in the questionnaires. If employees started consuming these healthy syrups instead of for instance water, this could have contributed to the observed difference in consumption of sugary drinks in favor of the control condition. Another factor to consider is the relatively high lifestyle score at baseline in this study population, with a mean of 7.1 on a scale from 0 to 10. Additionally, the response rate of only 12.8% suggests a potential selection bias. Although, it should be noted that some of the 1,400 employees that were approached, might not be eligible to participate, which indicates that the response rate could be somewhat higher. However, this information was not available to the researchers.

As reported by other studies, it is likely that employees who are already interested in health promotion or who are more health-conscious are more inclined to participate [54, 55]. The effectiveness of the integrated WHPP, as implemented by the participating organizations, might be affected by this, i.e., the relatively high lifestyle score at baseline and selection bias. As employees who are health-conscious may have already integrated these healthy behaviors into their lifestyle, independent of the integrated WHPP, which implies a potential ceiling effect [54]. Including employees with a less healthy lifestyle is a common and difficult to overcome challenge. Perhaps when working on your lifestyle is a generally accepted part of a normal working day, the threshold to participate would be lower for employees. In order to do so, it might help to create support among the whole organization about the importance of healthy lifestyle (at work). This can be done by engaging ambassadors (enthusiastic employees) or an active role of higher management (in emphasizing the importance of the topic) [11, 56–58]. Moreover, a targeted approach, tailored to the group of interest (‘difficult-to-reach-employees’) might by helpful [14]. In line with that, future interventions may benefit from adopting participatory approaches to enhance relevance, engagement, and impact.

Just as other similar interventions [21, 48, 59], the integrated WHPP under study was implemented in a higher income country within the European context. Results can therefore not be generalized to lower income countries or non-European contexts.

The study design, a C-RCT, mitigates the risk of contamination between the intervention and control conditions, which can be seen as a strength of the study. Nevertheless, as employees sometimes work from multiple locations, it is impossible to completely avoid contamination. Hence, not all activities were exclusively accessible for participants in the intervention condition; in some cases, participants in the control condition could also participate in implemented activities. As exact data about which employees work at multiple locations are lacking, the extent to which this affects the results cannot be estimated.

## Conclusions

Based on the current study, it can be concluded that the integrated WHPP, as implemented by the participating organizations, was not effective. Neither in improving the overall lifestyle nor separate health behaviors of employees. The limited exposure of employees to mostly minor and adapted activities, which did not fully meet the integrated WHPP criteria, combined with high baseline lifestyle scores, heterogeneous implementation across organizations, and potential issues with statistical power (partially due to the COVID-19 pandemic), should all be taken into account when interpreting these findings. The effectiveness of the integrated WHPP could not be conclusively demonstrated under the observed conditions of partial implementation. Future studies including more time to implement the integrated WHPP and a focus on continuity and more substantial activities with a higher frequency are recommended.

## Supplementary Information

Supplementary Material 1.

Supplementary Material 2.

Supplementary Material 3.

Supplementary Material 4.

Supplementary Material 5.

Supplementary Material 6.

## Data Availability

The datasets generated and analyzed during the current study are not publicly available due to some of the data being potentially identifiable. These data are available from the corresponding author on reasonable request.

## Abbreviations

WHPP: Workplace health promotion program
LTR: Dutch Trial Register (in Dutch: landelijk trial register)
LWHPN: Lombardy Workplace Health Promotion Network
C-RCT: Cluster randomized controlled trial
SLIQ: Simple Lifestyle Indicator Questionnaire
SQUASH: Short QUestionnaire to ASsess Health-enhancing physical activity
LPA: Light physical activity
MPA: Moderate physical activity
VPA: Vigorous physical activity
DASS-21: Depression Anxiety and Stress Scale
SWING: Survey Work-home Interference Nijmegen
MOS-SS: Medical Outcomes Study Sleep scale
GEE: generalized estimating equation
