# Supplementary material for: Effectiveness of an integrated approach for workplace health promotion on lifestyle of employees: results of a cluster randomized controlled trial
Source: BMC Public Health. 2025 Oct 14;25:3475. doi: 10.1186/s12889-025-24522-1 (PMC12523133; doi:10.1186/s12889-025-24522-1)
Supplement: Supplementary file 1 — Supplementary Material 1. [file 12889_2025_24522_MOESM1_ESM.pdf]

Additional file 1

Characteristics of the participating organizations

Organization	Occupational sector	Number of employees	Locations
1	Educational organization (university of applied science)	319[a]	2
2	Assurance, tax and consulting organization	639	7
3	Educational organization (middle school)	197	4
4	Retail organization	256[a]	27[b]

[a] Number of employees in participating departments within the organization. [b] Divided over four regional clusters.
