# Supplementary material for: Effectiveness of an integrated approach for workplace health promotion on lifestyle of employees: results of a cluster randomized controlled trial
Source: BMC Public Health. 2025 Oct 14;25:3475. doi: 10.1186/s12889-025-24522-1 (PMC12523133; doi:10.1186/s12889-025-24522-1)
Supplement: Supplementary file 2 — Supplementary Material 2. [file 12889_2025_24522_MOESM2_ESM.pdf]

Additional file 2

Consort checklist, extension for cluster designs

Section/topic and item No	Standard checklist item	Extension for cluster designs	Page No*
**Title and abstract**			
1a	Identification as a randomised trial in the title	Identification as a cluster randomised trial in the title	Title page
1b	Structured summary of trial design, methods, results, and conclusions (for specific guidance see CONSORT for abstracts)[11 12]		Page 1
**Introduction**			
Background and objectives:			
2a	Scientific background and explanation of rationale	Rationale for using a cluster design	Methods (page 4 - 5)
2b	Specific objectives or hypotheses	Whether objectives pertain to the cluster level, the individual participant level, or both	Page 2
**Methods**			
Trial design:			
3a	Description of trial design (such as parallel, factorial) including allocation ratio	Definition of cluster and description of how the design features apply to the clusters	Page 3 - 5
3b	Important changes to methods after trial commencement (such as eligibility criteria), with reasons		n.a.
Participants:			
4a	Eligibility criteria for participants	Eligibility criteria for clusters	Page 3 - 4
4b	Settings and locations where the data were collected		Page 6
Interventions:			
5	The interventions for each group with sufficient details to allow replication, including how	Whether interventions pertain to the cluster level, the individual participant level, or both	Page 4 - 5

Section/topic and item No	Standard checklist item	Extension for cluster designs	Page No*
	and when they were actually administered		
Outcomes:			
6a	Completely defined prespecified primary and secondary outcome measures, including how and when they were assessed	Whether outcome measures pertain to the cluster level, the individual participant level, or both	Page 6
6b	Any changes to trial outcomes after the trial commenced, with reasons		n.a.
Sample size:			
7a	How sample size was determined	Method of calculation, number of clusters(s) (and whether equal or unequal cluster sizes are assumed), cluster size, a coefficient of intracluster correlation (ICC or *k*), and an indication of its uncertainty	Page 4 – 5 (details are provided in a protocol paper)
7b	When applicable, explanation of any interim analyses and stopping guidelines		n.a.
**Randomisation**			
Sequence generation:			
8a	Method used to generate the random allocation sequence		Page 4 - 5
8b	Type of randomisation; details of any restriction (such as blocking and block size)	Details of stratification or matching if used	Page 4 - 5
Allocation concealment mechanism:			
9	Mechanism used to implement the random allocation sequence (such as sequentially numbered containers), describing any steps taken to conceal the sequence until interventions were assigned	Specification that allocation was based on clusters rather than individuals and whether allocation concealment (if any) was at the cluster level, the individual participant level, or both	Page 4 - 5
Implementation:			

Section/topic and item No	Standard checklist item	Extension for cluster designs	Page No*
10	Who generated the random allocation sequence, who enrolled participants, and who assigned participants to interventions	Replaced by 10a, 10b, and 10c	
10a		Who generated the random allocation sequence, who enrolled clusters, and who assigned clusters to interventions	Page 4 - 5
10b		Mechanism by which individual participants were included in clusters for the purposes of the trial (such as complete enumeration, random sampling)	Page 4 - 5
10c		From whom consent was sought (representatives of the cluster, or individual cluster members, or both) and whether consent was sought before or after randomisation	Page 3 - 4
Blinding:			
11a	If done, who was blinded after assignment to interventions (for example, participants, care providers, those assessing outcomes) and how		Page 4 - 5
11b	If relevant, description of the similarity of interventions		n.a.
Statistical methods:			
12a	Statistical methods used to compare groups for primary and secondary outcomes	How clustering was taken into account	Page 8
12b	Methods for additional analyses, such as subgroup analyses and adjusted analyses		Page 8
**Results**			

Section/topic and item No	Standard checklist item	Extension for cluster designs	Page No*
Participant flow (a diagram is strongly recommended):			
13a	For each group, the numbers of participants who were randomly assigned, received intended treatment, and were analysed for the primary outcome	For each group, the numbers of clusters that were randomly assigned, received intended treatment, and were analysed for the primary outcome	Figure 1 (page 4)
13b	For each group, losses and exclusions after randomisation, together with reasons	For each group, losses and exclusions for both clusters and individual cluster members	Figure 1 (page 4)
Recruitment:			
14a	Dates defining the periods of recruitment and follow-up		Page 3
14b	Why the trial ended or was stopped		Page 3
Baseline data:			
15	A table showing baseline demographic and clinical characteristics for each group	Baseline characteristics for the individual and cluster levels as applicable for each group	Table 1 (page 9)
Numbers analysed:			
16	For each group, number of participants (denominator) included in each analysis and whether the analysis was by original assigned groups	For each group, number of clusters included in each analysis	Figure 1 (page 4)
Outcomes and estimation:			
17a	For each primary and secondary outcome, results for each group, and the estimated effect size and its precision (such as 95% confidence interval)	Results at the individual or cluster level as applicable and a coefficient of intracluster correlation (ICC or *k*) for each primary outcome	Table 3 and table 4 (page 9)
17b	For binary outcomes, presentation of both absolute and relative effect sizes is recommended		
Ancillary analyses:			

Section/topic and item No	Standard checklist item	Extension for cluster designs	Page No*
18	Results of any other analyses performed, including subgroup analyses and adjusted analyses, distinguishing prespecified from exploratory		Supplementary information 2 and 3
Harms:			
19	All important harms or unintended effects in each group (for specific guidance see CONSORT for harms106)		n.a.
**Discussion**			
Limitations:			
20	Trial limitations, addressing sources of potential bias, imprecision, and, if relevant, multiplicity of analyses		Page 10 - 13
Generalisability:			
21	Generalisability (external validity, applicability) of the trial findings	Generalisability to clusters and/or individual participants (as relevant)	Page 10 - 13
Interpretation:			
22	Interpretation consistent with results, balancing benefits and harms, and considering other relevant evidence		Page 10 - 13
**Other information**			
Registration:			
23	Registration number and name of trial registry		Page 3
Protocol:			
24	Where the full trial protocol can be accessed, if available		Page 3
Funding:			
25	Sources of funding and other support (such as supply of drugs), role of funders		Title page

*Page numbers optional depending on journal requirements.
