# Supplementary material for: Effectiveness of an integrated approach for workplace health promotion on lifestyle of employees: results of a cluster randomized controlled trial
Source: BMC Public Health. 2025 Oct 14;25:3475. doi: 10.1186/s12889-025-24522-1 (PMC12523133; doi:10.1186/s12889-025-24522-1)
Supplement: Supplementary file 3 — Supplementary Material 3. [file 12889_2025_24522_MOESM3_ESM.docx]

**Additional file 3**

Examples of activities included in the catalogue

| **Health behavior** | **Individual level** | **Organizational level** |
| --- | --- | --- |
| Nutrition | Disseminate information about a healthy diet. For instance by videos made by employees or supervisors.^2^ | Adjustments to the company restaurant, such an increased variety of healthy selections and promoting only healthy products.^3^ |
| Physical activity | Provide employees with a logbook, pedometer, or activity tracker to monitor their goals.^1^ | Replace sitting desks with sit-stand desks. Make standing while working the norm (by keeping stand desks in the high position).^3^ |
| Mental balance | Provide individual or group sessions focused on stress management.^1^ | Create a designated area for silence.^3^ |
| Sleep | Provide workshops aimed at improving sleep.^1,2^ | Offer the opportunity for flexible and remote work, allowing employees to tailor their own work hours.^4^ |
| Smoking | Provide support for smoking cessation, such as referral to or advice from a professional, or access to a course or app.^1^ | Provide reimbursement for nicotine replacement products.^4^ |
| Alcohol | Provide support reducing excessive alcohol consumption, including referrals to or guidance from professionals, as well as access to courses or apps.^1^ | Organize alcohol-free social events, providing a range of 0.0% alternatives to alcoholic beverages.^3^ |

^1^Domain 1: Screening and support

^2^Domain 2: Information and education

^3^Domain 3: Adjustments in the social, digital or physical work environment

^4^Domain 4: Policy adjustments
