# Supplementary material for: Effectiveness of an integrated approach for workplace health promotion on lifestyle of employees: results of a cluster randomized controlled trial
Source: BMC Public Health. 2025 Oct 14;25:3475. doi: 10.1186/s12889-025-24522-1 (PMC12523133; doi:10.1186/s12889-025-24522-1)
Supplement: Supplementary file 4 — Supplementary Material 4. [file 12889_2025_24522_MOESM4_ESM.docx]

**Additional file 4**

Main findings of the process evaluation^1^

| **Implementation process component** | **Main findings** |
| --- | --- |
| Degree of implementation | Working groups were composed within each organization |
|  | Several activities were implemented by the organizations. But the criteria^2^ of the integrated WHPP were not met |
|  | Two out of four organizations conducted a needs assessment |
|  | All working groups used the catalogue to select activities |
| Perception of employees and employers | The integrated WHPP met the needs of one fifth of the employees |
|  | Almost half of the employees indicated that the workplace did not become healthier |
|  | More than one third of the employees felt involved in the implementation process |
|  | The working group was an essential and helpful component of the integrated WHPP |
|  | Supervisors need to find an adequate balance in stimulating participation in WHP |
| Contextual factors | A quarter of the employees did not have sufficient time to participate in implemented activities |
|  | Three quarters of the employees already had a healthy lifestyle |
|  | Working group members did not have sufficient time to implement activities |
|  | The absence of organizational policies regarding vitality was a barrier |
|  | An active and supportive role of higher management was sometimes lacking, but appears to be essential in the implementation process |

^1^Smit DJM, van Oostrom SH, Engels JA, Mooren-van der Meer S, Proper KI. The implementation of an integrated workplace health promotion program in Dutch organizations - A mixed methods process evaluation. PLoS One. 2024;19(11):e0308856. <https://doi.org/10.1371/journal.pone.0308856>

^2^Implementation of activities on both the individual and organizational level for at least two lifestyle themes
