# Supplementary material for: Effectiveness of an integrated approach for workplace health promotion on lifestyle of employees: results of a cluster randomized controlled trial
Source: BMC Public Health. 2025 Oct 14;25:3475. doi: 10.1186/s12889-025-24522-1 (PMC12523133; doi:10.1186/s12889-025-24522-1)
Supplement: Supplementary file 5 — Supplementary Material 5. [file 12889_2025_24522_MOESM5_ESM.docx]

**Additional file 5**

Drop-out analyses after six months of follow-up, baseline values and p-values of descriptive and outcome measures for control condition and intervention condition

| **Variable** | **Control condition** | | | **Intervention condition** | | |
| --- | --- | --- | --- | --- | --- | --- |
|  | **Mean/n(%) participants** | **Mean/n(%) dropouts** | **p-value** | **Mean/n(%) participants** | **Mean/n(%) dropouts** | **p-value** |
| **Sex (female)^a^** | 34 (51.5%) | 5 (29.4%) | 0.17 | 48 (58.5%) | 4 (50%) | 0.72 |
| **Age, years^b^** | 43.7 | 48.8 | 0.08 | 43.3 | 34.1 | 0.02 |
| **Educational level^a^** |  |  | 0.13 |  |  | 0.68 |
| ***Lower education*** | 3 (4.6%) | 0 (0.0%) |  | 6 (7.3%) | 0 (0.0%) |  |
| ***Moderate education*** | 22 (33.3%) | 2 (11.8%) |  | 23 (28.1%) | 1 (12.5%) |  |
| ***Higher education*** | 41 (62.1% | 15 (88.2%) |  | 53 (64.6%) | 7 (87.5%0 |  |
| **One or more chronic diseases^a, c, d^** | 25 (37.9%) | 11 (64.7%) | 0.06 | 25 (30.5%) | 6 (75%) | 0.02 |
| **Working hours per week^b, c^** | 35.7 | 35.5 | 0.94 | 35.7 | 37.9 | 0.23 |
| **Job intensity^a, c, e^** |  |  | 0.50 |  |  | 1.0 |
| ***Low physical load*** | 49 (74.3%) | 11 (64.7%) |  | 70 (85.4%) | 8 (100%) |  |
| ***Light physical load*** | 16 (24.2% | 6 (35.3%) |  | 7 (8.5%) | 0 (0.0%) |  |
| ***Moderate physical load*** | 1 (1.5%) | 0 (0.0%) |  | 5 (6.1%) | 0 (0.0%) |  |
| **Working from home^a^** |  |  | 0.86 |  |  | 1.0 |
| ***Fulltime*** | 2 (3.0%) | 0 (0.0%) |  | 3 (3.7%) | 0 (0.0%) |  |
| ***Parttime*** | 33 (50,0%) | 10 (58.8%) |  | 51 (62.2%) | 5 (62.5%) |  |
| ***Never*** | 31 (47.0%) | 7 (41.2%) |  | 28 (34.1%) | 3 (37.5%) |  |
| **Overall lifestyle^b^** | 6.9 | 7.4 | 0.34 | 7.2 | 8.0 | 0.12 |
| **LPA, minutes per week^b^** | 2347.1 | 1751.5 | 0.04 | 2479.0 | 2741.9 | 0.08 |
| **MPA, minutes per week^b^** | 569.4 | 665.8 | 0.52 | 462.6 | 273.1 | 0.06 |
| **VPA, minutes per week^b^** | 136.2 | 74.1 | 0.09 | 84.6 | 110.6 | 0.58 |
| **≥1 Sugary drinks per week^a^** | 35 (53.0%) | 9 (52.9%) | 1.0 | 39 (47.6%) | 4 (50%) | 1.0 |
| **Large snacks per week^a, f^** |  |  | 0.43 |  |  | 0.80 |
| ***0-2 per week*** | 23 (34.8%) | 9 (52.9%) |  | 40 (48.8%) | 4 (50.0%) |  |
| ***2-4 per week*** | 24 (36.4%) | 5 (29.4) |  | 20 (24.4%) | 1 (12.5%) |  |
| ***4-13 per week*** | 19 (28.8%) | 3 (17.7%) |  | 22 (26.8%) | 3 (37.5%) |  |
| **Small snacks per week^a, g^** |  |  | 0.94 |  |  | 0.53 |
| ***0-4 per week*** | 27 (40.9%) | 8 (47.1%) |  | 30 (36.6%) | 4 (50.0%) |  |
| ***4-6 per week*** | 16 (24.2%) | 3 (17.6%) |  | 25 (30.5%) | 3 (37.5%) |  |
| ***6-28 per week*** | 23 (34.9%) | 6 (35.3%) |  | 27 (32.9%) | 1 (12.5%) |  |
| **Perceived stress^a^** |  |  | 0.12 |  |  | 0.05 |
| ***Normal*** | 51 (77.3%) | 11 (64.7%) |  | 62 (75.6%) | 3 (37.5%) |  |
| ***Mild*** | 11 (16.7%) | 2 (11.8%) |  | 10 (12.2%) | 2 (25.0%) |  |
| ***Moderate to severe*** | 4 (6.0%) | 4 (23.5%) |  | 10 (12.2%) | 3 (37.5%) |  |
| **NFR^b^** | 29.6 | 33.7 | 0.66 | 34.7 | 73.9 | 0.008 |
| **Work-life balance^b^** | 0.8 | 1.0 | 0.11 | 0.8 | 1.3 | 0.19 |
| **Sleep disturbance^b^** | 25.6 | 22.8 | 0.45 | 29.9 | 38.8 | 0.36 |
| **Sleep somnolence^b^** | 22.4 | 19.6 | 0.44 | 21.4 | 28.3 | 0.23 |
| **Hours of sleep per night^b^** | 7.3 | 7.2 | 0.89 | 6.9 | 7.0 | 0.64 |
| **Smoking status (yes)^a^** | 10 (15.2%) | 2 (11.8%) | 1.0 | 1 (1.2%) | 0 (0%) | 1.0 |
| **Alcohol consumption per week^b^** | 4.3 | 4.3 | 1.0 | 4.2 | 2.9 | 0.28 |

A significance level of p < 0.002 with Bonferroni correction was applied to account for multiple comparisons. Abbreviations: LPA = Low physical activity, MPA = Moderate physical activity, VPA = Vigorous physical activity, NFR = Need for recovery.*indicates a significant difference between drop-outs and participants. ^a^ Fisher test was conducted to assess differences between participants and drop-outs. ^b^ t-test was conducted to assess differences between participants and drop-outs. ^c^ variable was not included in the analyses. ^d^ Self-reported physical or mental health problems. ^e^ Low physical load: A sedentary occupation. Light physical load: A standing occupation, including walking but no high intensity physical activity. Moderate physical load: An occupation that included occasional heavy lifting. ^f^ Large snacks: sweet, savory and fried. ^g^ Small snacks: Sweet and savory.
