# Supplementary material for: Effectiveness of an integrated approach for workplace health promotion on lifestyle of employees: results of a cluster randomized controlled trial
Source: BMC Public Health. 2025 Oct 14;25:3475. doi: 10.1186/s12889-025-24522-1 (PMC12523133; doi:10.1186/s12889-025-24522-1)
Supplement: Supplementary file 6 — Supplementary Material 6. [file 12889_2025_24522_MOESM6_ESM.docx]

**Additional file 6**

Drop-out analyses after twelve months of follow-up, baseline values and p-values of descriptive and outcome measures for control condition and intervention condition

| **Variable** | **Control condition** | | | **Intervention condition** | | |
| --- | --- | --- | --- | --- | --- | --- |
|  | **Mean/n participants** | **Mean/n dropouts** | **p-value** | **Mean/n participants** | **Mean/n dropouts** | **p-value** |
| **Sex, (female)^a^** | 28 (44.4%) | 11 (55.0%) | 0.45 | 38 (55.9%) | 14 (63.6%) | 0.62 |
| **Age, years^b^** | 45.1 | 43.4 | 0.58 | 42.6 | 42.4 | 0.94 |
| **Educational level^a^** |  |  | 0.42 |  |  | 0.43 |
| ***Lower education*** | 3 (4.8%) | 0 (0.0%) |  | 6 (8.8%) | 0 (0.0%) |  |
| ***Moderate education*** | 20 (31.7%) | 4 (20.0%) |  | 17 (25.0%) | 7 (31.8%) |  |
| ***Higher education*** | 40 (63.5%) | 16 (80.0%) |  | 45 (66.2%) | 15 (68.2%) |  |
| **One or more chronic diseases^a, c, d^** | 26 (41.3%) | 10 (50.0%) | 0.61 | 20 (29.4%) | 11 (50.0%) | 0.12 |
| **Working hours per week^b, c^** | 35.5 | 36.2 | 0.69 | 36.1 | 35.1 | 0.60 |
| **Job intensity^a, c, e^** |  |  | 0.36 |  |  | 0.87 |
| ***Low physical load*** | 46 (73.0%) | 14 (70.0%) |  | 58 (85.3%) | 20 (91.0%) |  |
| ***Light physical load*** | 17 (27.0%) | 5 (25.0%) |  | 6 (8.8%) | 1 (4.5%) |  |
| ***Moderate physical load*** | 0 (0.0%) | 1 (5.0%) |  | 4 (5.9%) | 1 (4.5%) |  |
| **Working from home^a^** |  |  | 0.89 |  |  | 0.66 |
| ***Fulltime*** | 2 (3.2%) | 0 (0.0%) |  | 3 (4.4%) | 0 (0.0%) |  |
| ***Parttime*** | 33 (52.4%) | 10 (50.0%) |  | 43 (63.2%) | 13 (59.1%) |  |
| ***Never*** | 28 (44.4%) | 10 (50.0%) |  | 22 (32.4%) | 9 (40.9%) |  |
| **Overall lifestyle^b^** | 7.0 | 7.2 | 0.54 | 7.3 | 7.0 | 0.58 |
| **LPA, minutes per week^b^** | 2375.2 | 1752.5 | 0.03 | 2495.7 | 2523.0 | 0.89 |
| **MPA, minutes per week^b^** | 531.4 | 771.2 | 0.19 | 430.8 | 492.0 | 0.73 |
| **VPA, minutes per week^b^** | 130.0 | 102.8 | 0.45 | 82.7 | 99.8 | 0.55 |
| **≥1 Sugary drinks per week^a^** | 26 (41.3%) | 13 (65.0%) | 0.08 | 33 (48.5%) | 14 (63.6%) | 0.23 |
| **Large snacks per week^a, f^** |  |  | 0.07 |  |  | 0.39 |
| ***0-2 per week*** | 21 (33.3%) | 11 (55.0%) |  | 33 (48.5%) | 11 (50%) |  |
| ***2-4 per week*** | 26 (41.3%) | 3 (15.0%) |  | 18 (26.5%) | 3 (13.6%) |  |
| ***4-13 per week*** | 16 (25.4%) | 6 (30.0%) |  | 17 (25.0%) | 8 (36.4%) |  |
| **Small snacks per week^a, g^** |  |  | 0.49 |  |  | 0.27 |
| ***0-4 per week*** | 26 (41.3%) | 9 (45.0%) |  | 23 (33.8%) | 11 (50.0%) |  |
| ***4-6 per week*** | 13 (20.6%) | 6 (30.0%) |  | 24 (35.3%) | 4 (18.2%) |  |
| ***6-28 per week*** | 24 (38.1%) | 5 (25.0%) |  | 21 (30.9%) | 7 (31.8%) |  |
| **Perceived stress^a^** |  | 0.46 |  |  |  | 0.86 |
| ***Normal*** | 49 (77.8%) | 13 (65.0%) |  | 50 (73.6%) | 15 (68.2%) |  |
| ***Mild*** | 9 (14.3%) | 4 (20.0%) |  | 9 (13.2%) | 3 (13.6%) |  |
| ***Moderate to severe*** | 5 (7.9%) | 3 (15.0%) |  | 9 (13.2%) | 4 (18.2%) |  |
| **NFR^b^** | 27.8 | 38.6 | 0.24 | 33.4 | 52.9 | 0.03 |
| **Work-life balance^b^** | 0.8 | 1.1 | 0.12 | 0.8 | 1.0 | 0.18 |
| **Sleep disturbance^b^** | 24.6 | 26.3 | 0.66 | 30.1 | 32.3 | 0.67 |
| **Sleep somnolence^b^** | 22.1 | 21.0 | 0.76 | 21.4 | 23.9 | 0.47 |
| **Hours of sleep per night^b^** | 7.2 | 7.3 | 0.73 | 6.9 | 6.9 | 0.98 |
| **Smoking status (yes)^a^** | 8 (12.7%) | 4 (20.0%) | 0.47 | 1 (1.5%) | 0 (0.0%) | 1.0 |
| **Alcohol consumption per week^a^** | 4.0 | 5.1 | 0.39 | 4.2 | 3.8 | 0.74 |

A significance level of p < 0.002 with Bonferroni correction was applied to account for multiple comparisons. Abbreviations: LPA = Low physical activity, MPA = Moderate physical activity, VPA = Vigorous physical activity, NFR = Need for recovery.*indicates a significant difference between drop-outs and participants. ^a^ Fisher test was conducted to assess differences between participants and drop-outs. ^b^ t-test was conducted to assess differences between participants and drop-outs. ^c^ variable was not included in the analyses. ^d^ Self-reported physical or mental health problems. ^e^ Low physical load: A sedentary occupation. Light physical load: A standing occupation, including walking but no high intensity physical activity. Moderate physical load: An occupation that included occasional heavy lifting. ^f^ Large snacks: sweet, savory and fried. ^g^ Small snacks: Sweet and savory.
